# Supplementary material for: Predicting superconducting transition temperature through advanced machine learning and innovative feature engineering
Source: Sci Rep. 2024 Feb 17;14:3965. doi: 10.1038/s41598-024-54440-y (PMC10874381; doi:10.1038/s41598-024-54440-y)
Supplement: Supplementary file 1 — Supplementary Information. [file 41598_2024_54440_MOESM1_ESM.docx]

**Supplementary Information**

**Predicting Superconducting Transition Temperature through Advanced Machine Learning and Innovative Feature Engineering**

Hassan Gashmard, Hamideh Shakeripour, Mojtaba Alaei

*Department of Physics, Isfahan University of Technology, Isfahan 84156-83111, Iran*

Corresponding author: Hamideh Shakeripour (hshakeri@iut.ac.ir)

## A) Jabir Package for Generating the Feature Space

## In order to generate features for superconducting materials, we designed and developed the Python language package called Jabir, which is capable of generating 322 atomic features for each compound. This package is also applicable to all materials, not just for superconducting materials.

## Jabir package calculates eight statistical relationships (such as standard deviation and variance) for each atomic feature (such as thermal conductivity) based on three types of Elements, Subscript and Fraction. The workflow of the Jabir package is depicted in Fig. 5 of the manuscript.

## As mentioned, atomic features are generated based on three types of Elements, Subscript and Fraction. The meaning of atomic features based on the Fraction is that the atomic features are multiplied by the fraction of that element in the compound. Similarly, the meaning of atomic features based on the Subscript is that the atomic features are multiplied by the subscript of that element in the compound. Finally, the meaning of atomic features based on Element is that atomic features are not multiplied by anything and just consider elemental values. In other words, each element is multiplied by one, regardless of fraction or subscript. Table 1 of the manuscript, briefly, explains the process used for calculating the mean thermal conductivity of the Mg_0.9_Fe_0.1_B_2_ compound, for instance.

We endeavored to incorporate novel and creative ideas into the design of the Jabir package. We must show the difference among the compounds clearly. Our objective is to extract more information from each compound. To achieve this, we consider three components: Element, Subscript, and Fraction. This approach is employed to provide a more comprehensive understanding of each compound.

Regarding the description of atomic features, we briefly explain all the most significant features which are shown in **Fig. 7** of the manuscript, in the following:

**range_Thermal_conduct_Elemental:** This feature computes the range of the thermal conductivity of atoms of each compound, considering their Elements.

**Sum_Subscript:** This feature computes the summation of subscripts of each compound.

**median_ElecAffinity_fraction:** This feature computes the median of the electron affinity of atoms of each compound, considering their Fraction.

**min_Pettifor_fraction:** This feature computes the minimum of the Pettifor number of atoms of each compound, considering their Fraction.

**max_ElecAffinity_fraction:** This feature computes the maximum of the electron affinity of atoms of each compound, considering their Fraction.

**range_dipole_polarizability_fraction:** This feature computes the range of the dipole polarizability of atoms of each compound, considering their Fraction.

**max_heat_formation_fraction:** This feature computes the maximum of the heat formation of atoms of each compound, considering their Fraction.

**min_ElecAffinity_fraction:** This feature computes the minimum of the electron affinity of atoms of each compound, considering their Fraction.

**max_ElecGativ_fraction:** This feature computes the maximum of the electronegativity of atoms of each compound, considering their Fraction.

**median_Thermal_conduct_fraction:** This feature computes the median of the thermal conductivity of atoms of each compound, considering their Fraction.

**max_heat_formation_Elemental:** This feature computes the maximum of the heat formation of atoms of each compound, considering their Elements.

**max_UnpairedElec_Elemental:** This feature computes the maximum of the number of unpaired electrons of atoms of each compound, considering their Elements.

**range_heat_formation_Elemental:** This feature computes the range of the heat formation of atoms of each compound, considering their Elements.

**mean_Pettifor_fraction:** This feature computes the mean of the Pettifor number of atoms of each compound, considering their Fraction.

**mean_heat_formation_fraction:** This feature computes the mean of the heat formation of atoms of each compound, considering their Fraction.

**range_ElecAffinity_Elemental:** This feature computes the range of the electron affinity of atoms of each compound, considering their Elements.

**range_Pettifor_Elemental:** This feature computes the range of the Pettifor number of atoms of each compound, considering their Elements.

**median_ElecGativ_Elemental:** This feature computes the median of the electronegativity of atoms of each compound, considering their Elements.

**mean_First_Ionis_Energy_Elemental:** This feature computes the mean of the first ionisation energy of atoms of each compound, considering their Elements.

**range_dipole_polarizability_Elemental:** This feature computes the range of the dipole polarizability of atoms of each compound, considering their Elements.

**median_dipole_polarizability_Elemental:** This feature computes the median of the dipole polarizability of atoms of each compound, considering their Elements.

**min_Ionic_Radius_Elemental:** This feature computes the minimum of the ionic radius of atoms of each compound, considering their Elements.

**median_NumElecValence_Elemental:** This feature computes the median of the number of valence electrons of atoms of each compound, considering their Elements.

**range_Ionic_Radius_Elemental:** This feature computes the range of the ionic radius of atoms of each compound, considering their Elements.

**min_ElecAffinity_Elemental:** This feature computes the minimum of the electron affinity of atoms of each compound, considering their Elements.

**max_Ionic_Radius_Elemental:** This feature computes the maximum of the ionic radius of atoms of each compound, considering their Elements.

**max_period_Elemental:** This feature computes the maximum of the period number of atoms of each compound, considering their Elements.

**min_Electric_Conduct_Elemental:** This feature computes the minimum of the electrical conductivity of atoms of each compound, considering their Elements.

**Number_Elements:** This feature computes the number of elements in each compound.

**range_period_Elemental:** This feature computes the range of the period number of atoms of each compound, considering their Elements.
